# Exosomes in Prostate Cancer: Putting Together the Pieces of a Puzzle

**DOI:** 10.3390/cancers5041522

**Published:** 2013-11-11

**Authors:** Carolina Soekmadji, Pamela J. Russell, Colleen C. Nelson

**Affiliations:** Australian Prostate Cancer Research Centre-Queensland, Institute of Health and Biomedical Innovation, Queensland University of Technology, Translational Research Institute, Level 3 West, 37 Kent Street, Brisbane, Queensland 4102, Australia; E-Mails: pamela.russell@qut.edu.au (P.J.R.); colleen.nelson@qut.edu.au (C.C.N.)

**Keywords:** exosomes, exosome biogenesis, extracellular vesicle, prostate cancer, biomarker, androgen receptor

## Abstract

Exosomes have been shown to act as mediators for cell to cell communication and as a potential source of biomarkers for many diseases, including prostate cancer. Exosomes are nanosized vesicles secreted by cells and consist of proteins normally found in multivesicular bodies, RNA, DNA and lipids. As a potential source of biomarkers, exosomes have attracted considerable attention, as their protein content resembles that of their cells of origin, even though it is noted that the proteins, miRNAs and lipids found in the exosomes are not a reflective stoichiometric sampling of the contents from the parent cells. While the biogenesis of exosomes in dendritic cells and platelets has been extensively characterized, much less is known about the biogenesis of exosomes in cancer cells. An understanding of the processes involved in prostate cancer will help to further elucidate the role of exosomes and other extracellular vesicles in prostate cancer progression and metastasis. There are few methodologies available for general isolation of exosomes, however validation of those methodologies is necessary to study the role of exosomal-derived biomarkers in various diseases. In this review, we discuss “exosomes” as a member of the family of extracellular vesicles and their potential to provide candidate biomarkers for prostate cancer.

## 1. Introduction

Cells secrete many different kinds of vesicles, termed extracellular vesicles (EV). The term EV covers a broad range of secreted vesicles, including exosomes, microvesicles, shed particles, apoptotic vesicles and apoptotic bodies. This has led to some controversy as to the exact definition of exosomes, and in this review we refer to exosomes based on their biogenesis, as nanosized vesicles which bud intracellularly at multivesicular endosomes, also called multivesicular bodies (MVB), while the other EV members are thought to bud directly from the plasma membrane [1,2,3].

Exosome biogenesis has been fundamental to understanding and defining what exactly is an exosome. As a product of intracellular budding from MVB, the exosomes consist of recycled proteins and lipids normally found in late endosomes [4]. Exosomes are also reported to contain nucleotides: RNAs such as miRNA, mRNA and tRNA, as well as DNA [5,6]. The presence of DNA in exosomes is interesting as the exosome is known to bud from the endosomal compartment, quite separate from the nucleus. 

The RNA species found in exosomes are reported to have biologically active functions in cells. In 2006, Ratajczak *et al.* showed that mRNA are found in murine embryonic stem cell small vesicles and that these vesicles are functional [7]. Valadi *et al.* then showed that both miRNA and mRNA are found in exosomes and that the exosomal mRNA could be translated in an *in vitro* model [5]. A further study from the same group showed that siRNA could be delivered via exosomes into target cells [8], confirming the potential relevance of exosomes as an RNA shuttle mechanism between cells [9,10,11]. The use of mRNA in exosomes was exploited further by Skog *et al.* to genotype EGFRvIII from nano-sized vesicles derived from serum of patients with glioblastoma tumors [12]. Altogether, the exosome is now accepted as an important mediator for cell to cell communication.

While there is agreement on the role of exosomes, the terminology to define whether vesicles belong to the exosome family currently relies heavily on morphology, size and purification methods used for their isolation. Most findings have focused on vesicles sized less than 100 nm in diameter as exosomes, while others employ a cut off value 150 or 200 nm diameter. This has become an important issue to address as many reports use the term, “microvesicles” instead of exosomes [13], “exosome-like vesicles” [14], or “prostasomes” [15,16], some of which in reality are likely to be a subset of the exosome family. Databases such as Exocarta and Vesiclepedia have emerged to help address this issue [17,18], however it remains largely unresolved as the data entered in these databases relies on the terminology used by authors in their published papers. 

## 2. Exosomes, Does Size Matter?

While it is widely accepted that exosomes are extracellular vesicles originating from MVB [3,19], there are discrepancies in reports about their size. In most published literature, exosomes are described as secreted vesicles with a 40–100 nm diameter [1]. Some publications use the term microvesicles, especially when the size may not fit into the currently accepted range for exosomes. A recent report on discussions from the 2012 meeting of International Society for Extracellular Vesicles (ISEV) also argued with the 100 nm cut off value for exosomes [20], leading to the question: can a vesicle be called an exosome when the diameter is greater than 100 nm?

Exosomes and microvesicles are not the only type of vesicles secreted by cells. Some agree that the term microvesicle covers a broader range of vesicles with a diameter from 100 nm up to 1 µm [5]. However, unlike the MVB origin of exosomes, microvesicles originate from the plasma membrane of eukaryotic cells after stimulation or apoptosis. Microvesicle biogenesis involves phospholipid distribution and dynamic contraction of the plasma membrane by cytoskeletal actin/myosin, regulated by ADP-ribosylation factor 6 (ARF6) [13,21]. Microvesicles are also sometimes called microparticles or “ectosomes” [3]. The 100 nm cut off value for microvesicles is also debatable as noted in the 2012 ISEV meeting that there is a lack of evidence that microvesicles that originate from the plasma membrane should be over 100 nm in diameter [20]. Other such as exosome-like vesicles, viral-like particles, microparticles, apoptotic bodies, and apoptotic vesicles have also been reported. Microparticles, viral-like particles and apoptotic bodies also originate from the plasma membrane. While microvesicles are formed after lipid exchange of inner and outer leaflets of cell membrane; the biogenesis of plasma membrane derived viral-like particles does not involve lipid exchange [3,22,23]. The apoptotic bodies, as the name indicates, are released during apoptosis, and contain DNA, histones, organelles and surface markers which allow them to be recognized by phagocytic cells [4,24]. As apoptotic bodies are usually bigger than exosomes, this type of vesicle would normally be separated from exosomes by a 10,000 g spin during stepwise ultracentrifugation. However, some other vesicles are around the same size as exosomes [3], and thus could become an issue during exosome purification.

Earlier published data on reticulocyte-derived exosomes show an electron microscopy image of a group of tiny dense vesicles containing transferrin receptor (TfR) around 50 nm in diameter, secreted through a ~200 nm pore [2,25]. The process of exosome secretion in reticulocytes is an essential process required for the secretion of TfR during maturation. Purification of platelet-derived exosomes has also reported exosomes of diameter around 40–100 nm, while larger vesicles can be obtained by a 10,000 g spin with diameters between 100 nm to 1 µm [26]. A recent report by Tauro *et al.* shows that exosomes purified using density gradients are more homogenous, with diameters around 100 nm, in comparison with exosomes purified by stepwise ultracentrifugation [27]. This is in agreement with discussions during the 2012 ISEV meeting that purification methods using density gradient centrifugation can enrich for a specific population of vesicles [20]. However this does not negate that exosomes could be bigger than 100 nm in diameter.

These perceived differences in the size of exosomes have made them problematic to categorise and for reporting on the actual vesicles being studied. Exosomes isolated from cancer cell lines, including prostate cancer cells (discussed in detail in Section 4), have diameters up to 150 nm [16,28]. Previously, comparative studies to isolate “pure” exosomes have been reported [27,29]; the isolation included steps such as exosome immunoprecipitation using CD9 antibody or pre-filtration using 0.2 µm cut off filters to help achieve the isolation of “pure” exosomes [28]. In particular, samples from clinical settings are even more difficult to process in order to obtain pure exosomes. A study of urine derived “microvesicles” from PCa patients, shows an electron microscopy image of >100 nm vesicles labeled with CD63 [30], a commonly accepted exosome marker. Vesicles derived from colorectal cancer ascites were also reported as microvesicles, due to their heterogenous vesicle size up to 200 nm, despite multiple purification steps including density gradient ultracentrifugation, sucrose gradient and finally using an OptiPrep gradient [31]. As the vesicles expressed proteins previously reported as exosomal markers such as Alix, TSG101, and CD81, it is arguable that these vesicles were exosomes. 

Others reported that exosomes isolated from synovial fluid by density gradient centrifugation were up to 300 nm in diameter [32]. Recent report by Otrowski *et al.* shows that the size of exosomes is in fact regulated through specific machinery driven by Rab27a, a member of Rab family of small GTPases in HeLa B6H4 tumour cell line. SiRNA of Rab27a increases the size of exosomes derived, while its isoform, Rab27b alters the localization of MVB [31]. The following report from the same group, show that the role of both Rab27a and Rab27b differ based on cell type. Unlike the study in Hela cells, in TS/A and 4T1 cells, shRab27a reduced exosome secretion while shRab27b altered the content of some exosomal proteins [33]. These reports challenge the current definition of exosomes based on size. Given that the cell type influences the characteristic of secreted exosomes, it is important to understand the exosome biogenesis in those cells before making a decision on what methodology would be optimal for isolating exosomes.

## 3. Exosome Identification: Fitting Heterogeneity into a Single Category

Up to date pitfalls of current available isolation methodologies have been discussed in the scientific community and recently have been reported in detail. They range from classical methodologies such as ultracentrifugation, as well as immunoprecipitation, gel-filtration, size exclusion chromatography, size exclusion filters, to microfluidic devices [20]. Identification is probably less problematic than purification as a few proteins are widely accepted as exosomal markers including: Alix, TSG101 (component of the Endosomal Sorting Complex Required for Transport, ESCRTs), CD9, CD63, CD81, and TfR [14,34,35,36] (Table 1). As mentioned above, depending on their cellular ancestry, exosomes carry cell-type-specific proteins. For example exosomes derived from oligodendrocytes contain myelin proteins whilst exosomes isolated from antigen-presenting cells contain the major histocompatibility complex (MHC) [4,37].

**Table 1:** Currently accepted exosomal protein markers and their common subcellular localization.

Marker	Subcellular localisation	REFs
Alix	cytosol	[38]
TSG101	cytosol	[39]
CD9	plasma membrane	[40,41]
CD63	plasma membrane and cytosol	[42,43]
CD81	plasma membrane	[40]
TfR	perinuclear or plasma membrane	[44]

The Alix (PDCD6IP) interacts with the TSG101 and CHMP4 components of the ESCRT-I and III complexes, and these interactions also mediate HIV viral budding [45,46]. TSG101 binds to HRS, a protein involved in sorting ubiquitinated cargo to ESCRT-I, which is then recruited to ESCRT-II and then ESCRT-III [47,48,49]. Alix is also associated with endocytic and signal transduction proteins such as endophilins and CIN85 (SH3KBP1), and structural proteins of the cytoskeleton, including actin and tubulins, which confirms that Alix is involved in regulating the MVB membrane dynamic. Additionally, recent studies into exosome biogenesis show that Alix can interact with syndecan and syntenin, and this interaction regulates the endosomal membrane budding which then leads to an increase in syntenin-contained exosome biogenesis [50,51,52,53]. The syntenin can be recruited to the plasma membrane through binding with the cytosolic domain of syndecans [47], suggesting that the concerted Alix-syndecan-syntenin interaction drives the secretion of exosomes. 

Some reports have used different markers to identify exosomes, such as the tetraspanins, CD9 and CD81. CD9 and CD81, however, are also commonly found at the plasma membrane [40,41]. CD9 has been shown to interact with CD63, which is found in the MVB [26], in activated platelets and both interact with CD81 in human melanoma [54,55]. The CD9 and CD63 are both members of transmembrane 4 superfamily (TM4SF), a heterogeneous membrane-bound glycoprotein [56]. The TM4SF members can form multicomponent structures and these complexes associate with beta-1 integrin, contributing towards cell motility [57]. CD63 is also involved in cancer progression and has been suggested as a predictive biomarker for some cancers [58,59,60]. Increases in CD9 synthesis in response to 12-O-Tetradecanoylphorbol 13-Acetate (TPA), a PKC nonspecific activator, was found to increase the level of CD9 found in exosomes [34] whilst secretion of exosomes from cells in CD9 knockout mice was reduced. CD9 expression increases beta catenin secretion through exosomes which then negatively regulate Wnt signaling pathways [61], suggesting that CD9 may be involved in the signaling and recruitment of proteins into exosomes. 

Electron microscopy images have confirmed the heterogenous nature of exosome populations, as not all visualized exosomes contain one particular marker. For example, immunogold labeling using CD63 antibody was only found on approximately 50% of platelet derived exosomes (from 500 vesicles isolated by a combination of 10,000 ×*g* pre-spin, followed by density gradient centrifugation) [26]. Recently, immunoprecipitation was proposed as an alternative method to isolate pure exosomes. The CD9 and CD63 antibodies have been used for selective isolation of exosomes [28,62]. However this technique will require some understanding on whether these exosome surface markers are expressed by the cell lines being investigated. Recently, Baietti *et al.* reported that CD63 exosomes also contain syntenin [53], indicating that the CD63 exosomes are the same population as the Alix-syntenin exosomes. Furthermore, over expression of CD63 further decreased the exosomal yields of syntenin and Alix [53], suggesting the involvement of CD63 in the biogenesis of Alix positive exosomes. 

The exosome pathways shows a significant similarity with HIV viral particle in T cells, reported by Gould *et al.*, in that these vesicles are 50–100 nm vesicles enriched in classical markers CD63 and CD81. As such they were classified as exosomes [63]. The electron microscopy images show that the viral associated vesicles bud from lipid raft-enriched plasma membrane, with similar density on sucrose gradient and size by electron microscopy [63,64]. The HIV retroviral biogenesis, similar to exosomes, requires TSG101 and the AAA^+^ ATPase VPS4 [63]. The interactions of VPS4 and tetrapeptide PSAP motif of an accessory protein, arrestin domain-containing protein 1 (ARRDC1), are required for the scission of the narrow membrane neck at the plasma membrane, which mediates viral particle budding [64]. These reports show that exosome markers are important regulators in the process of exosome and retroviral particle biogenesis. Whilst some of these markers are found commonly at the plasma membrane, they have been shown to interact with each other during these processes and that even though the molecular players are the same, the exosome biogenesis process can vary depending on cellular origin. 

Exosome biogenesis has been shown to also be mediated without the involvement of the ESCRT complex. A study by Trajkovic *et al.* shows that sphingolipid ceramide in a mouse oligodendroglial cell line, Oli neu cells, is required to transfer the exosome associated domain into endosomal lumen which is thus involved in selecting the cargo of the exosome [65]. This process seems to be solely dependent on lipid processing as TSG101 and Alix have no roles in this process. Sphingomyelinases, enzymes generating ceramide from sphingomyelin, have been shown to be involved in the biogenesis of such exosomes in glial cells. Higher order oligomerization (oligomerization of oligomers), can play a role in exosome biogenesis, possibly by mediating membrane curvature at the plasma membrane which allows vesicle secretion [66].

**Figure 1:**
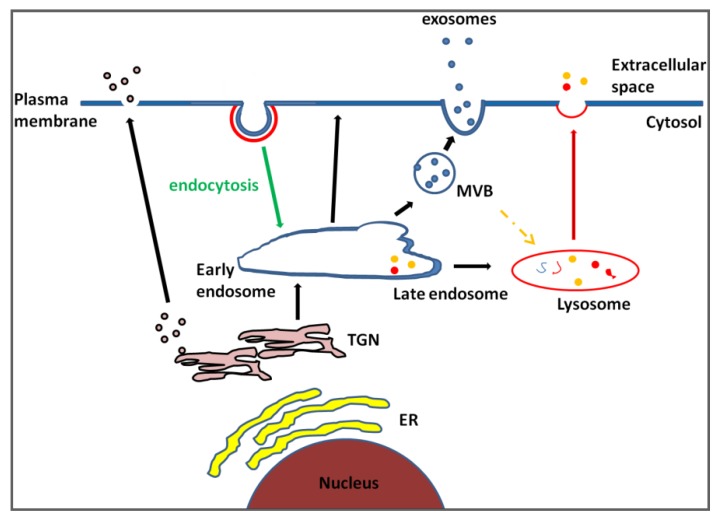
Schematic representation of exosome biogenesis. Extracellular molecules are endocytosed through various endocytic pathways such as clathrin mediated endocytosis or lipid rafts, and trafficked to early endosomes. The cargo and molecules are then recycled back to the plasma membrane; transferred to lysosome for degradation or secreted through exosome pathways. Evidence shows that the biogenesis of exosomes is mediated by intraluminal membrane budding at the Multi Vesicular Bodies (MVB) which are formed through an endosome maturation process. Both MVB and lysosome have been shown to be capable of fusing with the plasma membrane and releasing their contents, and some studies have highlighted the ability of MVB to fuse with the lysosome [1,73,74]. Classical soluble protein secretory pathways from Trans Golgi network (TGN) are also indicated. ER: endoplasmic reticulum.

The heterogenous nature of exosomes was recently confirmed by Bobrie *et al.*, who showed that separate populations of exosomes, expressing milk fat globule—epidermal growth factor—factor 8 (Mfge8; previously identified as especially enriched in dendritic cell exosomes) or CD9 vesicles could both be obtained from conditioned medium of 4T1 cells by ultracentrifugation at 10,000 ×*g*, whereas CD63, Alix, TSG101 and Hsc70 were present at high levels only after the 100,000 ×*g* spin [67]. The CD63, Alix, TSG101 and Hsc70 were also shown to be undetectable in 100,000 ×*g* pellet in Rab27a impaired cells, while CD9 remained [68]. In a subsequent study, it was shown that increasing the time of flotation of vesicle pellets on a sucrose gradient (by 100,000 ×*g* spin) for up to 62 h resulted in finding exosome markers CD63, CD9 and Mfge8 in different density fractions [68], confirming the different population of exosomes. These studies beg the question as to which marker would then be the best to confirm that isolated vesicles are exosomes. In dysregulated diseases such as cancer, alteration of exosome biogenesis pathways is likely as the cancer progresses, which may alter the role and expression of what are currently used as exosomal markers. Additionally, each cell type may express some but not all of the exosomal markers, thus understanding the pathway(s) involved in exosome biogenesis in the relevant diseases is necessary (see the schematic diagram of exosome biogenesis in Figure 1). 

## 4. Do Exosomes Provide Potential Prostate Cancer Biomarkers?

Currently there is a great interest in understanding the role of exosomes in cancer progression, including prostate cancer (PCa) and whether exosomes could be used as a potential source of biomarkers in PCa [28,30,69]. Until now, Prostate Specific Antigen (PSA) has been commonly used for PCa detection and to monitor tumour recurrence and progression. PSA is secreted by PCa cells and can be found in blood plasma. However, PSA is not always reliable as a PCa biomarker and levels can be elevated for non-cancerous reasons [70]. Clinical trials have also shown it is difficult to determine the baseline for PSA values to exclude the presence of PCa completely [71].

Hence better or additional biomarker(s) are required for PCa prognosis. Apart from indicating tumour progression, there is no study that describes a biomarker that can indicate treatment response. Patients who have a positive PCa biopsy of high grade (Gleason score 7 or higher) are typically treated by surgery or radiation therapy. In contemporary series up to 25% of these patients will suffer a relapse within 10 years and undergo androgen deprivation therapy (ADT) [72]. While this offers temporary cancer control, most patients ultimately develop castrate resistant prostate cancer (CRPC) with a rise in serum levels of prostate specific antigen (PSA), used as a marker of tumor growth, and continue to progress with metastatic disease [72]. 

Men with advanced PCa vary in their responses to ADT. This treatment strategy blocks androgen synthesis or inhibits the Androgen Receptor (AR). Androgens bind and activate AR and regulate a large set of responsive genes that control growth, stress, proliferation, differentiation, and cell survival [75,76,77]. Drugs designed to block the AR signalling axis, are either directly blocking synthesis of testosterone (T), or acting as agonists or antagonists of the luteinizing hormone-releasing hormone (LH-RH) secretion by the hypothalamus which leads to suppression of follicle stimulating hormone (FSH) and T [78]. 

As a marker of tumour growth, PSA is an androgen regulated gene. PSA is not found in the exosomes isolated from serum of xenograft-bearing mice, indicating that PSA is secreted into the extracellular space in a soluble form [28]. As ADT represses AR activation, this consequently represses PSA expression compromising measurement of treatment response. Exosomes, which are secreted through the late endosome-MVB pathway, may be able to replace or complement the PSA measurements to determine PCa patient prognosis, especially for patients undergoing ADT or chemotherapy drug treatment. The discovery of biomarkers for treatment resistance is possible by isolating exosomes from a longitudinal sample collection from patients in weekly or monthly intervals prior to, during and after drug treatment. This collection would allow scientists to monitor the changes in exosomal biomarker profiles for drug-treated patients.

Studies of exosomes relating to PCa have also been confused with terminology, in this case, “prostasomes”. The prostasomes are vesicles isolated from semen. Stridsberg *et al*. reported the prostasomes as neuroendocrine-like vesicles, as they contain neuroendocrine markers, chromogranin B, neuropeptide Y, and vasoactive intestinal polypeptide [79]. Prostasomes have a mean diameter of 150 nm, even though some authors report a broader range of 40–500 nm vesicles; they contain CD38, PSMA, and Anx1, have a complex membrane composition (cholesterol/phospholipid ratio 2:1), as well as expressing exosomal markers, CD9 and CD63 [6,80,81,82], and other markers such as CD46, CD55 and CD59 [83,84,85]. Prostasome specific markers are commonly found to be located at the plasma membrane (Table 2). 

**Table 2:** Prostasomes protein markers and their common subcellular localization.

Marker	Subcellular localisation	REFs
PSMA	plasma membrane and cytosol	[86]
CD38	plasma membrane	[87]
Anx1	intracellular granule	[88]
CD59	plasma membrane	[89]
CD46	plasma membrane	[89,90]
CD55	plasma membrane	[89]

Images of prostasomes show a multiplicity of vesicular structures, with smaller vesicles inside larger vesicles. The prostasomes have a trilamellar membrane structure, are round or egg shaped and some have “cauliflower-like” protrusions. The prostasomes also vary in electron density [82]. This description is significantly different from exosomes, which are usually described as round, cup-shaped vesicles. However, nanosized vesicles secreted by PC3 cells, have also been previously reported as prostasomes, presumably due to the discrepancy in vesicle size as discussed above. PC3 derived vesicles have a diameter of 30–150 nm with a round, cup shape morphology and they contain exosome markers TSG101 and CD63 [16,91]. In a paper by Llorente *et al.*, EM images of secreted vesicles, some with diameters around 150 nm, and the CD63 (one of many exosome markers)-labelled nanovesicles were found in organelles that resemble the MVB. Llorente *et al.* have also shown an apparent release of these vesicles [91]. While the paper named the vesicles as “microvesicles”, the MVB origin of these vesicles suggests that these vesicles are exosomes and that exosomes from PC3 can be bigger than 100 nm. Another prostate cancer paper shows an EM image of isolated exosome secreted from the PC346C labeled with CD9 which is also bigger than 100 nm [28]. 

Validation of exosomes in the provision of PCa biomarkers can only be achieved by understanding the exosome biogenesis in PCa. Some have suggested that vesicles isolated from biofluids should be termed extracellular vesicles since their biogenesis is unknown. However, publications to date call these exosomes based on their purification methodology, markers and size. Given the current literature, we continue to use the term “exosomes” for vesicles that harbour currently accepted exosome specific markers. Proteins isolated from circulating exosomes are not reflective of the proteins that are highly expressed in the respective cells, or in other cells as well as PCa cells [16,28,92], demonstrating that the content of exosomes is selectively screened intracellularly from cytoplasmic proteins through the MVB sorting process [2]. This process of exosome biogenesis provides a source of biomarkers that do not rely on a single protein or molecule, but a group of proteins, RNAs, DNAs and lipids in a population of vesicles, all of which in combination may help to stage heterogeneous diseases such as PCa. As drugs used to treat cancer would also affect the intracellular dynamic, the exosomes may also provide improved biomarkers to indicate treatment responsiveness or resistance.

### 4.1. Exosomal Proteins in Prostate Cancer

Exosomes can be isolated from human blood plasma and from urine. Exosomes are stable at low pH as found in urine where no changes in their content were observed for at least 18 h at 37 °C; this provides an advantage for clinical studies [93]. Proteomic studies of PCa exosomes have been reported on various PCa cell lines and clinical samples. PCa related exosomes from clinical samples in general show the presence of some cancer related proteins such as CD9, CD81, and TSG101, Annexin A2, Fatty Acid Synthase (FASN) and a PCa specific biomarker, FOLH1 (Prostate Specific Membrane Antigen or PSMA) [94,95]. PSMA is a transmembrane glycoprotein that is expressed in normal prostatic epithelial cells and elevated in androgen deprived PCa, and confirmed to be highly upregulated in poorly differentiated, metastatic, and hormone refractory carcinomas [86,96]. Quantitative reverse transcriptase-polymerase chain reaction (qRT-PCR) assays with primers specific for PSMA have been shown to be more effective than PSA-specific primers in detecting PCa cells [97]. PSMA was currently utilized as an immunoscintigraphic target using the antibody conjugate ProstaScint to detect occult PCa, but the antibody used for this detection could only recognized an internal epitope; as such it would detect dead PCa cells. New tests have been developed using an antibody against an external epitope [98]. PSMA is also used in immunotherapy of PCa [99]. PSMA expression has been evaluated as predictive marker of prostate-specific antigen (PSA) recurrence; even though PSMA is independently associated with PSA recurrence in a high-risk cohort [96,100]. Mitchell *et al.* have reported a pilot study (10 PCa patients and 10 healthy donors) in which five of eight PCa patients had PSMA in their urine derived exosomes, whilst three of eight PCa patients expressed PSA in the exosomes as indicated by western blot analysis [93]. The presence of PSA in exosomes from this study might suggest that the exosome isolation procedure from urine may be contaminated by soluble proteins as it has been reported Jansen *et al.* [28], that PSA is not found in the exosomes.

PCa proliferation and survival are greatly influenced by PI3K pathways. In PCa, loss of PTEN, a lipid phosphatase and negative regulator of PI3K, will cause a more severe disease, as hemizygous mutation of PTEN increases the risk and biochemical relapse, while homozygous deletion of PTEN increase the incidence of metastasis of PCa [101]. Recently, Gabriel *et al.* reported that PTEN is found in exosomes isolated from DU145 cells and these exosomes can influence cell proliferation in PTEN negative cells such as DU145Kd (knock down PTEN DU145 cells) and PC3. PTEN is also found in exosomes isolated from the blood of PCa patients, while normal individuals have no PTEN in their exosomes. In this study however, PSA was also found in exosomes from both PCa patients and normal individuals [102]. 

In plasma from healthy donors, CD63-labelled nanosized vesicles were found to contain CD9, CD81, and class I and II MHC molecules, common exosomal markers [30,93]. Some reports indicate the trend of exosome markers in cancer progression. For example, in clinical studies, reduced expression levels of the tetraspanin CD9 are correlated with tumour progression in a range of cancers [103,104]. Crossing a CD92/2 (KO) murine model with TRAMP mice (a PCa mouse model showing de novo development of spontaneously metastasising PCa) shows that ablation of CD9 had no detectable effect on *de novo* primary tumour onset, but increased metastasis to the liver [105]. In small cell lung cancer cell lines, CD9 is highly expressed on cells resistant to cisplatin or etoposide [106], suggesting that CD9 is a possible marker to indicate treatment resistance.

Progression and proliferation of PCa, similar to other cancers, is influenced by p53 protein, which regulates the transcription of a variety of genes as a response to stress. p53 is accumulated in the cell nuclei in PCa and this localisation is associated with missense p53 mutations. Mutated p53 alleles are found in ~25% of advanced PCa, suggesting a role for p53 mutation in the progression of at least a subset of PCa [107,108]. Studies of the role of p53 in exosome secretion have been reported as p53 pathway enhances exosome secretion. However genes that are transcriptional targets of p53 are not found in exosomes after p53 activation in non-small lung cancer cell lines [109], showing the upstream regulation of p53 on exosome secretion process. Up to date, we have not found any study on the role of p53 in exosome secretion in PCa.

Comparison of exosomal proteins secreted by some AR positive (AR+) and AR negative (AR−) PCa cell lines grown in serum free medium has been reported. In this study, CD9, HSP90, and HSP70 were used as exosome markers. However, it was not very clear whether the exosome pathways of each PCa cell line studied were affected by the growth conditions used in the study. Three proteins, Heat shock protein 90 kDa beta member 1 (GRP94 or ENPL, known as an ER stress marker), Heat shock 70 kDa protein 5 (glucose regulated protein 78 kDa, GRP78, also an ER stress marker), and Annexin A2 pseudogene 2 (AXA2L) were found to be present specifically in AR− cell lines (DU145, PC3 cells), and two proteins Chaperonin containing TCP1 subunit 7 (TCPH) and Histone cluster 2 H2ab (H2A2B) were specifically found in AR+ PCa cell lines (VCaP, C4–2, LNCaP), and also in RWPE-1 (AR+ normal adult human prostate cells transfected with a single copy of the human papilloma virus 18 (HPV-18)) [65]. Other potential PCa biomarkers such as Prostate Stem Cell Antigen (PSCA) were found in exosomes from rat reticulocytes and urine exosomes [110,111,112]. Further studies to investigate whether these markers could also reflect AR action in clinical PCa are still needed. 

### 4.2. Exosomal RNA in Prostate Cancer

Apart from the protein content, the RNA species found in exosomes could also provide an indication for cancer progression. For example, despite the lack of PSA protein in exosomes, the RNA was present in exosomes isolated from both VCaP and PC346C (an androgen dependent cell) [28]. In PCa, specific gene fusions such as the *TMPRSS2*-*ERG* gene fusion event occur in a subset of patients and are associated with lethal PCa. *TMPRSS2*-*ERG* gene fusion is androgen regulated and found in 50% of clinically localised PCa, and in 90% of PCa over-expressing ERG [113,114,115]. The *TMPRSS2*-*ERG* RNA is found in exosomes isolated from VCaP (an androgen responsive cell) [28]. Both *TMPRSS2*-*ERG* and a PCa biomarker, Prostate Cancer Antigen (PCA-3) mRNA were also reported in urine derived exosomes (CD63-labelled vesicles) [30], showing the mRNA content of exosomes is informative and can provide potential biomarkers for PCa.

Micro RNA (miRNA) content in exosomes is also a potential biomarker for PCa. The feedback loop between miRNAs, their corepressors and AR has been shown in non-cancerous androgen-responsive tissues [116]. miRNAs are short, 19–25 nt RNAs with partial homology to sequences in their target mRNAs. Incorporation of miRNAs into the RNA-induced silencing complex (RISC) allows its binding to the 3'UTR target genes resulting in down-regulation of protein expression through translation repression, mRNA cleavage or mRNA destabilization [117,118,119], and repression of protein synthesis [120]. A protein involved in miRNA processing, argonaute 2 (AGO2), is membrane-associated and a member of RNA-induced silencing complex (RISC). The AGO-miRNA-mRNA complexes have been shown to be sorted into MVB, even though AGO2 does not end up in secreted exosomes, the associated mature miRNAs are selectively packaged into exosomes for secretion [121]. In PCa, knockout of the miRNA processing enzyme, DICER, has resulted in LNCaP cells becoming unresponsive to androgen. Knock-down of DICER in the LNCaP cell line also induced the expression of DICER corepressors, NCoR and SMRT [116], confirming that miRNA exosomes could be a potential source of biomarkers. Mitchell *et al.* have reported that expression of miR-141, the first miRNA reported as a potential diagnostic marker in PCa, correlates significantly with serum PSA levels and could detect individuals with advanced metastatic prostate cancer with 60% sensitivity and 100% specificity [122]. Others have characterized other miRNAs as PCa biomarkers from biofluids and have shown several promising candidates depending on the stage of PCa [122,123,124,125]. 

Identification of AR regulated miRNA has been reported using LNCaP and VCaP (both AR positive) cell lines. Seventeen miRNAs were reportedly up or down regulated by more than 1.5-fold upon DHT treatment. Additionally, 103 miRNAs were found to be differentially expressed in PC346 xenografts after the mice were castrated [126]. miR-10a showed similar androgen regulation in both cell lines and xenografts [126], and interestingly, was also found in exosomes from PC3 (an androgen independent cell line) [127], suggesting that miR-10a could be an exosome-derived miRNA biomarker for PCa irrespective of androgen action. 

Bryant *et al.* reported on 11 miRNAs derived from microvesicles which were present in a significantly higher amount in plasma samples of PCa patients compared to normal individuals. Ten miRNAs were also identified to differ quantitatively between patients with distant metastasis in comparison with normal individuals [123] (list of miRNA in Table 3). In this study, miR-375 and miR-141 were significantly increased in microvesicles from sera of metastatic patients compared with non-recurrent PCa patients. The microvesicles were isolated using 1.2 µm filter and concentrated using a filter concentration (150 kDa cut off). This purification strategy is not able to differentiate exosomes from other microvesicles, thus it is not clear whether the miRNAs studies were exosomally derived miRNAs. 

In cell line models, functional studies on miRNA exosomes using DU145 (AR negative) and CW22RV1 (AR positive) cell lines were recently published. The PCa cells were infected with exosomes derived from docetaxel resistant cells, and exosome-exposed cells became capable of conferring the resistance toward docetaxel [128]. Cells exposed to exosomes isolated from patients after docetaxel treatment have also shown an increased level of docetaxel resistance [128], indicating the potential of exosomes to indicate docetaxel resistance. However, the PCa CW22RV1 cells are known to be infected by murine derived XMRV virus. XMRV are known to be able to transfer their miRNA via viral entry mechanisms to infect various PCa cell lines [129]. As the XMRV virus produces viral particles with the same diameter as exosomes [130], this will require some caution on interpreting any studies using the CW22RV1 cell line. 

**Table 3:** Potential miRNA biomarkers associated with circulating vesicles in PCa.

Detected miRNAs	Sample	Cohort	REF
107, 130b, 141, 181a-2, 2110, 301a, 326, 331-3p, 432, 484, 574-3p, 625	Plasma	All PCa cases versus normal control individuals	[123]
107, 141, 181a-2, 2110, 301a, 326, 432, 484, 574-3p, 625	Plasma	Localised PCa versus normal control individuals	[123]
582-3p, 20a, 375, 200b, 379, 572, 513a-5p, 577, 23a, 1236, 609, 17, 619, 624, 198, 130b	Plasma	Localised prostate cancer versus metastatic PCa	[123]
107, 574-3p, 375, 200b, 141	Urine	Benign versus PCa	[123]

### 4.3. Exosomal Lipids in Prostate Cancer

Exosomes are enriched in glycosphingolipids, sphingomyelin, cholesterol, and phosphatidylserine in various PCa cell lines, irrespective of the presence of AR. Interestingly, the lipid species found to be enriched in exosomes, similar to proteins and miRNA, are also not the ones mostly found in cell lysates [131,132]. In PC3 cells, there is an 8-fold enrichment of lipids/mg protein in exosomes, in comparison with the cell lysates [132]. 

Evidence shows that exosomes are derived from MVB and that the MVB can translocate to the plasma membrane and release its exosome content. The late endosomes, from where MVB originate, contain a complex system of internal membranes that are involved in both the degradation and recycling of proteins and lipids [133] (see Figure 1). The connection between lipid dynamics and the exosome biogenesis pathway was reported; association of Alix and lysobisphosphatidic acid (LBPA)-containing liposomes was found to alter the MVB and late endosome membrane dynamics [134,135]. Disruption of LBPA is known to cause cholesterol accumulation in late endosomes in some cells [49]. The LBPA, together with Alix, regulates cholesterol levels in endosomes in the cholesterol storage disorder, Niemann-Pick type C (NPC) diseases. Reduced expression of Alix decreases both LBPA levels and cellular cholesterol levels. This phenotype is also reversible; it could be rescued by adding endogenous LBPA, suggesting that LBPA may control the cholesterol capacity in endosomes [136]. 

In what capacity does lipid play a role in exosome secretion in PCa? Some studies show the role of lipids is to influence the amount of exosomal proteins being secreted. As well a particular lipid species, the sphingolipid, has a role in exosome biogenesis (discussed in Section 3). Calveolin-1 (cav-1), an integral membrane protein which binds to lipid rafts, has been shown to be upregulated in metastatic PCa and its expression is correlated with Gleason Score [137], indicating the potential of cav-1 as biomarker for advanced PCa. Llorente *et al.* have reported that the amount of cav-1 in isolated exosomes from PC3 cells increases with time [16]. Treatment using membrane-impermeable, cholesterol-extracting drug (MBCD) increased cav-1 levels in exosomes, but not in the cell lysates [16], suggesting that the cholesterol/lipid raft is involved in regulating exosomal protein secretion. Cholesterol, however, does not seem to influence exosome size [16]. It has been noted by the authors however that MBCD does not only affect cholesterol [16], as it had previously been reported that MBCD may induce membrane alterations [138,139]. Evidence that lipids are involved in exosomal secretion pathways has also been demonstrated by the contribution of lipid rafts to the release of HSP70 from heat shocked epithelial cells [140]. HSP70 is commonly found in exosomes [131], suggesting that lipid rafts play a role in the content of secreted exosomes. However, another study reports that the secretion of HSP70 by heat shocked PBMCs is independent from lipid rafts [141]. Studies on the role of other lipid species such as sphingolipids and phospholipids on exosome secretion from PCa are needed.

## 5. Conclusions

Exosomes have been suggested as carriers of potential biomarkers in several diseases, including cancer. What is lacking at the moment is a comprehensive study of intracellular organelle dynamics in those diseases. A list of many proteins, lipids, and RNAs species needs to be put into a context of PCa, before defining which is or are the best biomarker(s) found in exosomes. 

As the EV field will continue to grow, it is now becoming clear that there is a need for a better validated isolation methodology for exosomes (see also Section 1; [20]). Exosome purification by immunoprecipitation seems to be desirable in the near future to purify secreted exosomes [27]. Density gradient or column separation has been shown to be capable of separating different vesicles; however, a study which investigates whether the intracellular exosomes of the respective cells or cell lines are also reflective of the isolated secreted vesicles is necessary. Otherwise, we may just eliminate important data by eliminating some vesicles which are currently considered too big to be exosomes. 

It is also noted that the use of miRNAs as biomarkers is limited by conflicting data between studies due to a lack of standardization in methodology [142]. Any single miRNA may not give diagnostic or prognostic accuracy, as increased levels of a particular miRNA can be associated with several different types of tumours [142]. This creates a niche for exosomes which have been shown to contain several miRNAs. Exosomes may indeed be beneficial to help stage a heterogeneous disease such as PCa. Longitudinal studies in PCa patients on various therapies will provide exosomes for serial analysis which may help to answer some of these questions.

As exosomes have been shown to be capable of mediating cell to cell communication, investigations on exosome biogenesis would not just cover the use of exosomes as biomarkers. In this case, current evidence shows that RNAs in exosomes are the strongest candidates for intercellular communications. It is not known whether all proteins and lipids in exosomes are biologically active. However, it would be interesting to determine whether proteins, RNAs and lipids, all work in concert to mediate the exosome function as a communication mediator between PCa cells. 
